# GFN1-xTB-Assisted Machine Learning for Electronic Structure Screening of Metal–Organic Frameworks

**DOI:** 10.1021/acs.jctc.6c00979

**Published:** 2026-08-11

**Authors:** Ashna Jose, Aron Walsh

**Affiliations:** Department of Materials, 4615 Imperial College London, London SW7 2AZ, U.K.

## Abstract

Metal–organic frameworks (MOFs) are versatile materials with tunable crystal structures, morphologies, and chemistries, offering diverse physical and chemical properties. Although typically electrically insulating, specific combinations of organic and inorganic components can impart electrical conductivity to MOFs. The virtually limitless chemical space of MOFs, however, presents a significant challenge in identifying optimal candidates for various applications. Although density functional theory (DFT) can probe their electronic structure, its high computational cost hinders the discovery of novel electroactive MOFs using machine learning due to limited data. To tackle these challenges, a semiempirical extended tight-binding approach (GFN1-xTB) is employed to compute the electronic properties of a dataset of MOFs, and it is shown that GFN1-xTB approximates MOF band gaps well, as compared to semilocal DFT. These data are used to train an interpretable Δ-learning model that predicts the difference between low- and high-fidelity band gaps, given by xTB and DFT data at the hybrid level, respectively. This model outperforms direct models trained using only the DFT values. The Δ-learning model also outperforms models with deep-learning architecture, fine-tuned on our custom dataset to predict the band gaps of MOFs. With limited high-quality DFT band gaps, taking advantage of Δ-learning using low-cost GFN1-xTB leads to better predictions than relying on DFT data alone.

## Graphical abstract

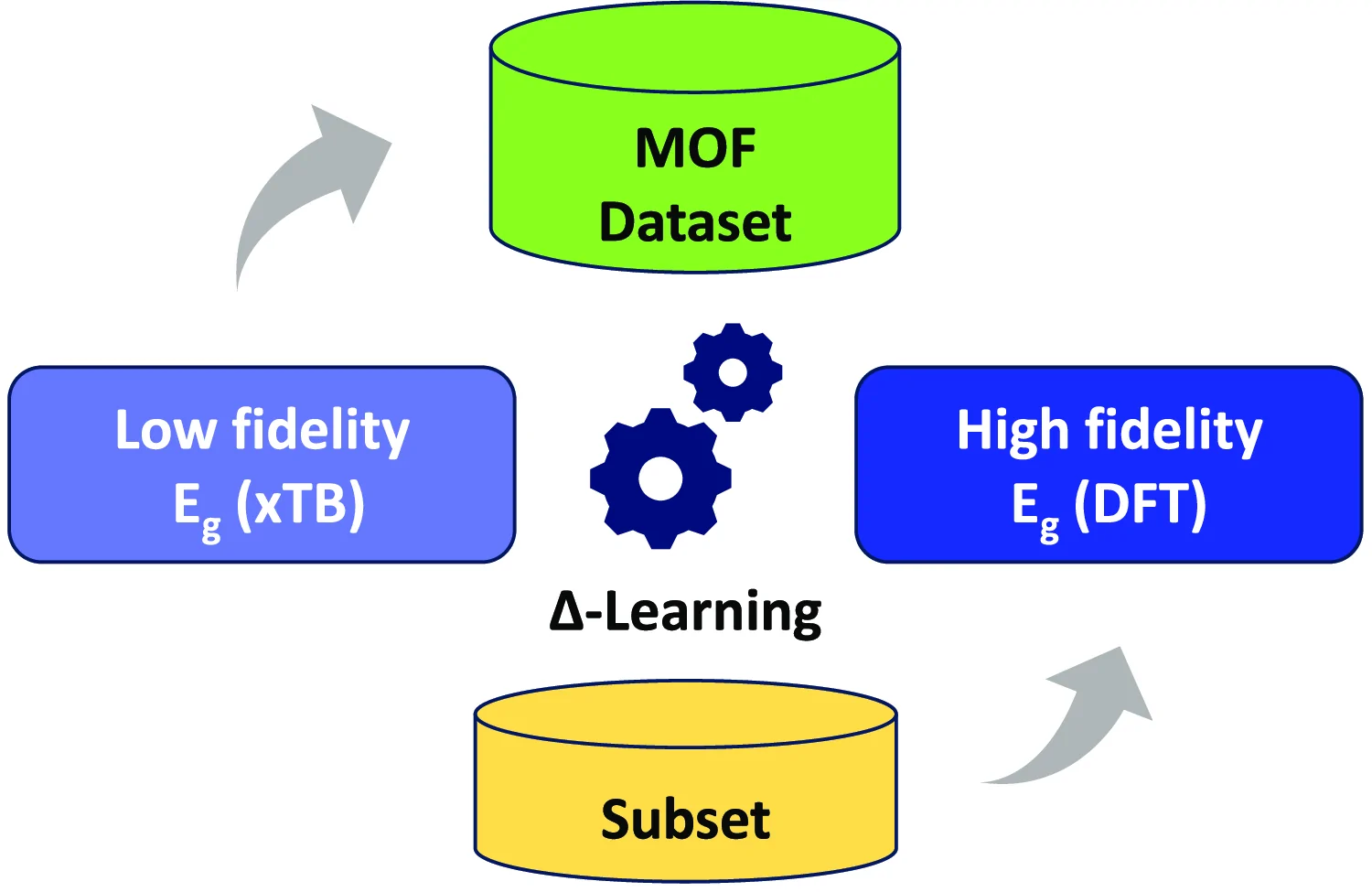

## Introduction

Metal–organic frameworks (MOFs)^1,2^ are formed through coordination bonds between metal ions and organic ligands. They show promise in a variety of applications,^3^ such as gas capture,^4,5^ energy storage,^6,7^ catalysis,^8,9^ and sensing.^10^ They have also been used for applications in electrochemistry,^11^ such as batteries,^12^ supercapacitors,^13^ and fuel cells.^14,15^ This versatility of MOFs is due to their highly porous and chemically tunable nature.^16^ Further, electronic properties of MOFs can be altered by introducing ions or guest molecules in their porous framework,^17,18^ which has fueled a growing interest in investigating these properties.^19–22^ More than 100,000 MOFs have been reported in the Cambridge Structural Database^23,24^ so far, along with various public datasets with synthesized and hypothetical MOFs.^25–28^ However, this vast design space makes the search for novel MOFs with specific properties challenging.

Computational techniques^29–32^ such as density functional theory (DFT)^33–37^ are often leveraged to screen these datasets, providing detailed insights into the electronic structure and energetics, although due to the high number of atoms in MOFs, this is computationally intensive. Particularly, using DFT at the hybrid level of theory is unfeasible to explore a substantial portion of the MOF design space. In recent years, artificial intelligence (AI) has been utilized to accelerate MOF discovery.^38–51^ These approaches significantly reduce computational cost by learning surrogate models that approximate DFT-level accuracy at a fraction of the expense. However, deep machine learning (ML) architectures^52–58^ are particularly well-suited for large datasets, and the lack of such datasets with high-quality DFT data is a significant bottleneck in advancing this field.

In this context, semiempirical methods such as the self-consistent charge density functional tight binding (SCC-DFTB^59,60^ ) balance accuracy and computational cost by approximating DFT through a Taylor expansion of the total energy around a reference electron density, significantly reducing computational cost by 2–3 orders of magnitude. However, its limited element coverage and low transferability restrict its applicability in high-throughput studies. Recently, Grimme et al. developed two extended tight-binding (xTB) methods for calculating structures, vibrational frequencies, and noncovalent interactions (GFN*n*, *n* = 1, 2)^61,62^ of large molecular systems. These methods include precomputed parameters for each atomic species, encompassing both global and element-specific parameters for most elements up to atomic number *Z* = 86. Thus, the total number of parameters are fixed, and no pairwise parameters are required, making it attractive for large-scale studies, while the broad parameter coverage significantly enhances the general applicability of the method across diverse systems. This approach has been employed to investigate a wide range of properties in a variety of materials.^63–68^ GFN*n*-xTB has also shown promise in accurate geometry optimization^69^ and in reliably approximating X-ray diffraction (XRD) patterns, textural characteristics, and electronic properties of two-dimensional (2D) and three-dimensional (3D) MOFs.^70,71^ These capabilities position GFN*n*-xTB as a practical tool for rapid high-throughput screening, balancing between computational efficiency and predictive accuracy.

In this work, GFN1-xTB is employed to compute electronic band gaps for a dataset of 4941 MOFs^51^ derived from the quantum MOF (QMOF) dataset.^27^ Band gaps computed with GFN1-xTB are found to be in agreement with those obtained using DFT (Perdew–Burke–Ernzerhof, PBE) for the majority of MOFs. Material descriptors^72,73^ are used to encode the MOFs and dimensionality reduction^74^ is used to analyze the data structure in its descriptor space. The analysis reveals that GFN1-xTB predicts the band gaps of MOFs with transition metals with low accuracy. Furthermore, Δ-learning^75^ using an XGBoost^76^ model is utilized to train the difference between band gaps computed with GFN1-xTB and DFT (PBE/HSE06). This model outperforms the XGBoost model trained directly on DFT data as targets, as well as deep learning models such as MOFTransformer^54^ and Crystal Graph Convolutional Neural Networks (CGCNN)^77^ that are commonly adopted to predict band gaps in complex systems. This demonstrates that using classical machine learning models, while exploiting low- and high-fidelity data, is an effective approach for MOFs, while also being computationally less intensive and more interpretable, leading to a better understanding of structure–property relationships. A schematic of the band gap prediction framework developed in this work is shown in Figure 1.

**Figure 1:**
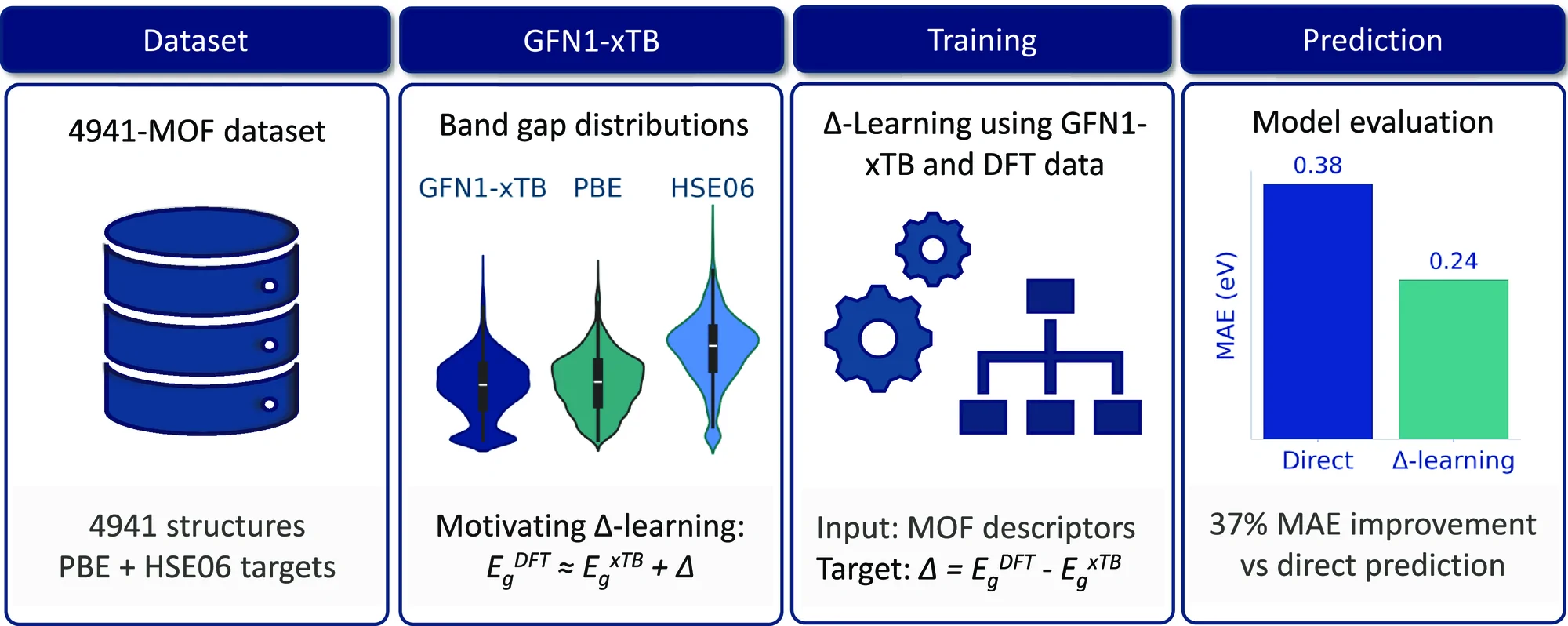
Schematic of the Δ-learning workflow for predicting DFT-level band gaps of metal–organic frameworks. Semiempirical GFN1-xTB band gaps are computed for a dataset of MOFs and used alongside descriptors to train a tree-based model, with the systematic xTB-to-DFT correction (Δ) as the target. The trained model predicts DFT-quality band gaps at a fraction of the computational cost, outperforming direct machine learning prediction.

## Computational Methods

### Geometry, Frequency, Noncovalent, eXtended Tight Binding (GFN-xTB)

The semiempirical GFN1-xTB^61^ approach employs minimal basis sets of atom-centered orbitals and considers only valence electrons in a linear combination of atomic orbitals (LCAO), including terms up to the third order in energy. Contrary to previous semiempirical methods,^60^ the repulsive term is not designed to reproduce the original term relative to the DFT contribution and to compensate for the approximations of other terms in DFT. Instead, it is parametrized more globally, which simplifies the formulation and enhances its applicability across a broader range of chemical systems. This eliminates the need to redefine the potential for each element pair, avoiding pairwise parameters. The D3 method^78^ is employed to compute the dispersion energy term. Slater-type orbitals are used to describe AOs and approximated through contractions of standard primitive Gaussian functions. Hydrogen bonding is described by a second s-function for hydrogen, and d-polarization functions for higher row elements are also included. With these parametrizations, GFN1-xTB comprises 16 global and about 1000 element-specific parameters, making it magnitudes faster than DFT, and a computationally inexpensive way to obtain large labeled datasets. This favorable balance between computational efficiency and accuracy makes it well-suited for high-throughput screening. Note that GFN2-xTB^62^ was tested preliminarily in this study but was not pursued further due to convergence failures, consistent with findings in previous work.^71,79,80^

### Δ-Learning and Descriptors

Δ-Learning^75^ is a machine learning approach in which a model is trained to predict corrections to target values, defined as the difference between high-fidelity references and their corresponding low-fidelity approximations. Rather than learning a direct mapping from a structure to target property, this strategy focuses on modeling the residual error, leading to improved data efficiency and potentially a better predictive accuracy. In this work, band gaps computed using GFN1-xTB, *E*_g,xTB_ are taken as the low-fidelity data, and DFT band gaps, *E*_g,DFT_, are considered as true target values. An XGBoost model^76^ is used to train Δ for each MOF, *i*, in the training set, and Δ_*i*_ is given by

1 $$\Delta_{i}=E_{g,{\mathrm{DFT}}_{i}}\left(x_{i}\right)-E_{g,{\mathrm{xTB}}_{i}}\left(x_{i}\right)$$

Here, *x*_*i*_ denotes the set of descriptors employed to encode the structure. Learning this correction becomes particularly beneficial when high-quality reference data are not readily available. By leveraging inexpensive calculations as a baseline, the model effectively learns from low-cost approximations, reducing the need for large volumes of computationally expensive DFT data. In such cases, Δ-learning from cheaper approximations of the targets can be leveraged to train ML models with higher accuracy, as was previously shown by Zhang et al.^81^ XGBoost models are employed in this work due to their strong predictive performance and interpretability. It also enables analysis of structure–property relationships and provides insights into feature importance, helping to identify the key factors influencing the model’s predictions. This interpretability is valuable in materials science applications, wherein understanding the underlying drivers of predictions is as important as achieving high predictive accuracy.

A concatenation of three descriptors is considered in this work: Stoichiometric-135 (ST-135),^82^ Revised Autocorrelations (RACs),^42,83^ and Atomic Property Weighted Radial Distribution Functions (APRDFs).^84^ These are commonly used to encode MOFs and ML models trained with these predict DFT band gaps accurately.^44^ ST-135 is composed of 118 features describing the elemental fractions along with 17 statistical attributes of elemental properties such as atomic number, radii, etc. RACs are products and differences of heuristic atomic properties in graphs. Metal-centered, linker, and functional group descriptors are generated weighted by atomic properties such as atom identity, connectivity, Pauling electronegativity, covalent radii, nuclear charge, and polarizability. Averaging over all of the atoms in each MOF produces 384 features. Finally, APRDFs use the weighted probability distribution of finding an atom pair in a spherical volume with radius *R* inside the unit cell to encode a structure. Atomic properties such as atomic mass, number, etc. are used to weigh the RDFs, leading to a feature length of 648. Concatenating all features produces a final feature vector for each MOF, with 1167 dimensions. A combination of these complementary descriptors allows the model to capture compositional, topological, and chemical information simultaneously, resulting in a more complete representation of the structures.

### Dataset and Technical Details

A subset of 4941 MOFs from the QMOF dataset^27^ curated in ref 51 was utilized in this work. DFTB+^85^ was used to compute band gaps using GFN1-xTB, starting from the optimized geometries obtained from the QMOF dataset. The structures were used to obtain the inputs of the DFTB+ code. The maximum angular momentum was set based on the elements in the structure using MaxAngularMomentum. The *k*-point mesh was generated automatically with the automatic_density function from pymatgen, and the target density was set to 2000. Once the calculations were completed, the band gaps were extracted from the outputs obtained from DFTB+. This set of calculations and analysis was automated using a custom Python code, openly available at https://github.com/AshnaJose/MOF-xTB.

XGBoost models with 200 estimators were trained on the training data. The learning rate was set to 0.1, and the maximum depth of the trees was set at 6. As the dimension of the feature space is large, feature selection was used to select the important features using the XGBoost feature selection function. This selection was performed with the training set. Three sets of feature selection were performed, with different target properties, i.e., band gaps obtained using GFN1-xTB, PBE, and HSE06. The top (important) features primarily comprised features from the RAC descriptor, along with a few from the ST-135 and the APRDF descriptor sets. This implies that RACs describe electronic properties, such as the band gap in this work, accurately. These results are summarized in Figure S1.

## Results

### GFN1-xTB Benchmarking

For the subset of 4941 MOFs, DFT band gaps at two levels of theory were obtained: PBE values from the QMOF dataset and high-fidelity HSE06 values from ref 51. To compute band gaps with GFN1-xTB, PBE-optimized structures from the QMOF dataset were used. xTB calculations were performed using DFTB+ ^85,86^ (automated by the custom Python code available publicly as described earlier). These calculations were successful for 4922 MOFs.

Figure 2a shows the parity plot using hex-bins comparing reference PBE band gaps to computed GFN1-xTB band gaps. The semiempirical method largely succeeds in capturing the electronic behavior of this dataset, with a mean absolute error (MAE) of 0.37 eV, along with the added advantage of being computationally inexpensive compared to DFT. Further, Figure 2b shows the comparison between the reference HSE06 band gaps and computed GFN1-xTB values. GFN1-xTB, in general, underestimates the HSE06 band gaps with an MAE of 1.26 eV. However, a clear correlation is observed between the two, which also resembles the known trend between PBE and HSE06 band gaps (MAE = 1.16 eV), as shown in Figure 2c for the 4941-dataset. This shows that GFN1-xTB predicts the electronic properties of MOFs reliably. These results demonstrate that GFN1-xTB serves as an effective method for approximating the electronic band gaps in MOFs. Error distributions of the GFN1-xTB band gaps relative to those of PBE and HSE06 are shown in Figure S2.

**Figure 2:**
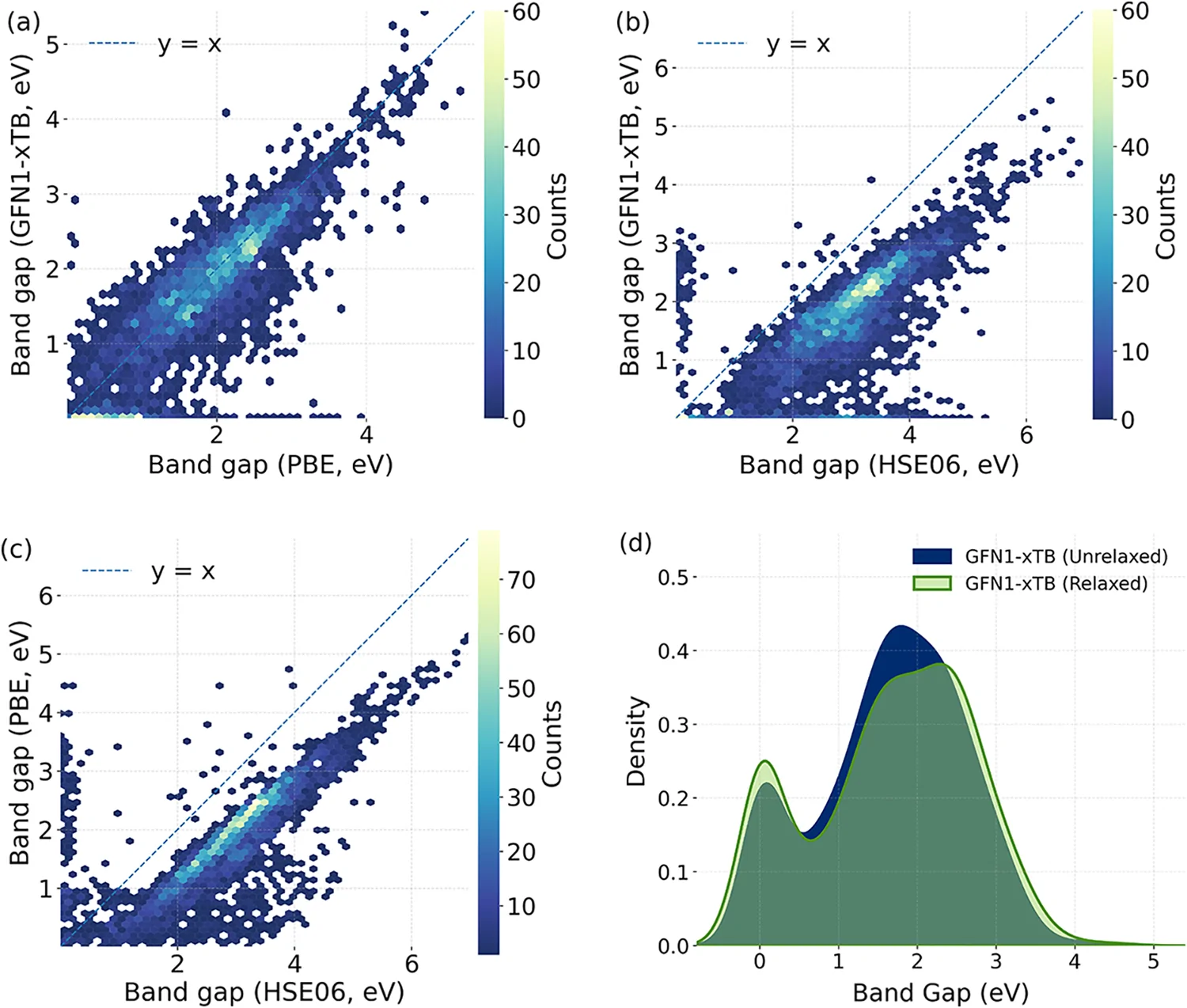
(a) Parity plots using hex-bins comparing the computed GFN1-xTB band gaps for the 4941-dataset with reference DFT band gaps at the PBE and (b) HSE06 levels. (c) Parity plot between DFT band gaps at the PBE and the HSE06 level of theory for reference. In (a)–(c), bin colors represent point density and the dashed line indicates *y* = *x*. (d) Band gap distributions of 500 MOFs obtained from unrelaxed and relaxed GFN1-xTB calculations using kernel density estimates (KDE) (resulting in the tail that extends below 0 eV).

As mentioned earlier, the xTB calculations were performed using the structures from the QMOF database directly, which were relaxed with DFT at the PBE­(D3) level, without relaxing again using GFN1-xTB. To investigate the effect of relaxation, we curated a subset of 500 MOFs, selected via a stratified sampling based on the PBE band gap values of the 4941-dataset, thereby preserving the overall distribution of electronic properties across the sampled subset. These structures were relaxed with GFN1-xTB, followed by computation of band gaps. The comparison of these results with the ones obtained earlier using the pre-relaxed structures is shown in Figure 2d for the subset of 500 MOFs. The kernel density estimate (KDE) distributions indicate that relaxation with GFN1-xTB underestimates the band gaps in some regions, while slightly overestimating in others, although the overall trend remains consistent. Because this study employs structures pre-relaxed with DFT, an additional relaxation step is of limited importance. However, for studies using an unrelaxed dataset, such as those of hypothetical MOFs, these results suggest that structural relaxation becomes significant, as it may lead to non-negligible changes in both geometry and the resulting electronic structure. We also performed this analysis on a chemically diverse subset of MOFs and additionally tested GFN1-xTB relaxation starting from unrelaxed experimental structures (taken from the CoRE MOF 2019 dataset^87^). In both cases, the band gap distributions remained consistent (see S3), confirming the robustness of our results.

### Chemical Trends in Band Gap Predictions

It is noteworthy that in both Figure 2a,b, a subset of MOFs exhibits high DFT band gap values while showing comparatively low band gaps when calculated with GFN1-xTB. Dimensionality reduction using *t*-distributed Stochastic Neighbor Embedding (*t*-SNE)^74^ was used to visualize the dataset in the ST-135 stoichiometric feature space, enabling identification of underlying patterns in high-dimensional compositional data. Figure 3a shows that *t*-SNE partitions the dataset into distinct clusters in this feature space. The ST-135 feature set separates the dataset into clusters based on the range of atomic number feature, which broadly indicates the metal center type in each MOF, as shown in Figure 3a. This feature, obtained from Matminer,^72^ is defined as the difference between the highest and lowest atomic numbers of the elements present in a given structure. Effectively, it reflects the element with the highest atomic number within the material, which is typically the metal in the case of MOFs. Thus, this clustering is primarily governed by variations in the metal center identity of MOFs.

**Figure 3:**
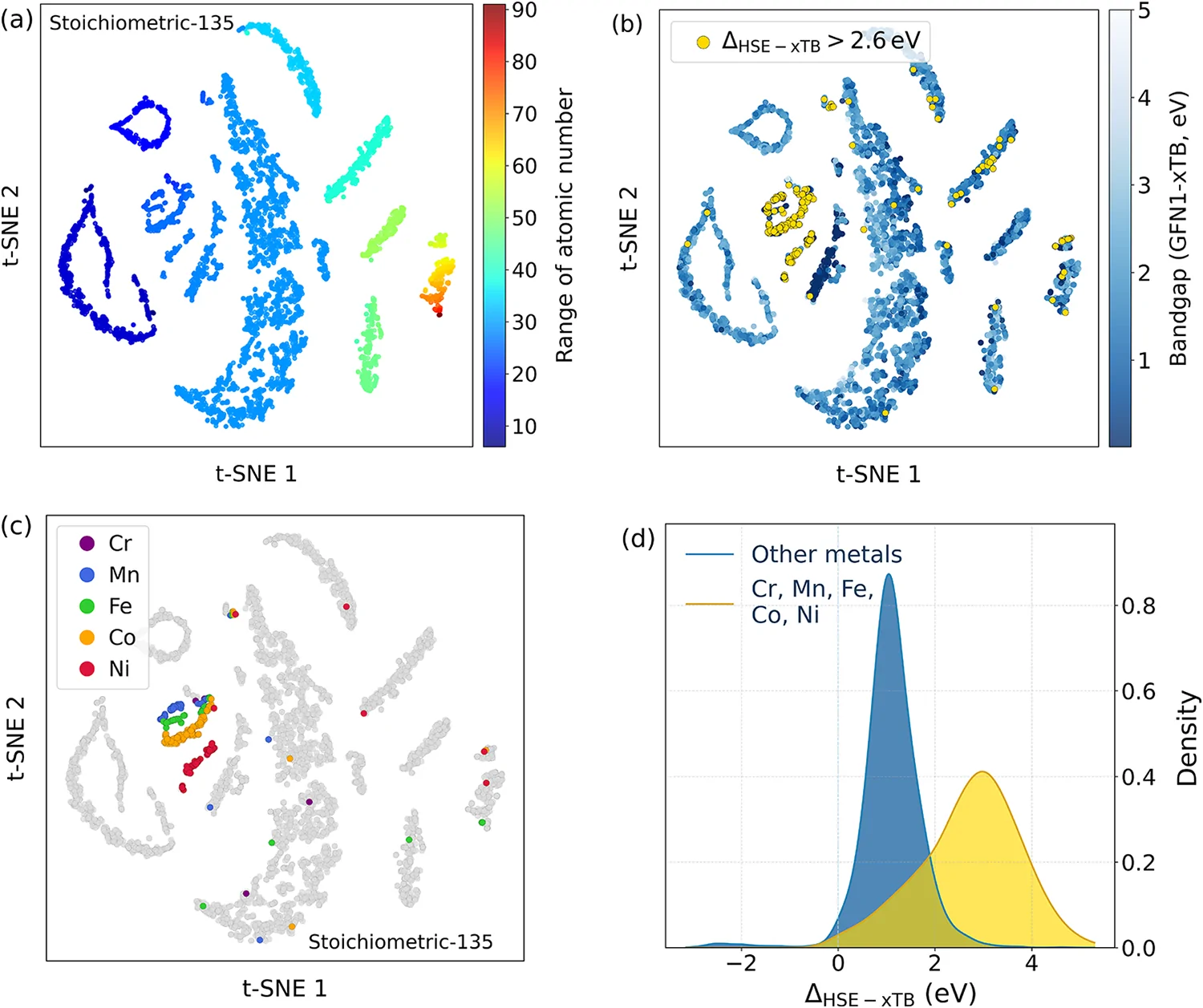
(a) *t*-SNE of the 4941-dataset in the ST-135 feature space. The colors represent the value of the range of atomic number in each structure, one of the features of ST-135. (b) *t*-SNE, highlighting GFN1-xTB band gap values for each MOF in shades of blue. MOFs with high band gap error (Δ_HSE‑xTB_ above the threshold) are shown in yellow. (c) *t*-SNE highlighting the clusters that correspond to MOFs with Cr, Mn, Fe, Co, and Ni. (d) Kernel density estimates of Δ_HSE‑xTB_ for MOFs containing Cr, Mn, Fe, Co, or Ni (yellow) versus all other MOFs (blue), showing the systematic underestimation of band gaps by GFN1-xTB, particularly for open-shell transition metal MOFs.

The *t*-SNE visualization also reveals that the 5% MOFs with the highest Δ_HSE‑xTB_ values (shown as Δ_HSE‑xTB_ > 2.6 eV in Figure 3b, where 2.6 eV is the top 5% MAE threshold), which are primarily the MOFs incorrectly predicted as highly conductive, are clustered in a small region of this descriptor space. Element-specific features from ST-135 reveal that these clusters correspond to MOFs that contain the transition metals Cr, Mn, Fe, Co, and Ni, as highlighted in Figure 3c. This implies that GFN1-xTB was unable to correctly approximate the electronic behavior of MOFs with transition metals, particularly due to the spin-restricted formulation of the method as employed in this work. The complex d-electron behavior of transition metals is also difficult to capture using semiempirical methods including GFN1-xTB due to the simplification of the self-consistency in DFT using parametrization. Furthermore, this implementation of GFN1-xTB is inadequate in describing strong correlation effects, which are prominent in materials with transition metals, and are captured by hybrid functionals and DFT + *U*. Finally, the magnetic coupling (ferro/antiferromagnetic) between metal centers may also influence band gaps, although in most MOFs, the metal centers are bridged by extended organic linkers, and therefore, intersite exchange is likely weak.

As a result of this, the predictive accuracy is lower for open-shell systems using the implementation used in this work. This is illustrated in Figure 3d, which shows the kernel density estimates (KDE) of the residuals Δ_HSE‑xTB_ = *E*_g,HSE06_ – *E*_g,xTB_ for MOFs containing Cr, Mn, Fe, Co, or Ni (in yellow) versus all other MOFs (in blue). The transition metal group exhibits a significantly broader and more positive residual distribution, indicating a systematic underestimation of band gaps by GFN1-xTB for these systems. However, for MOFs containing other metals, Δ_HSE‑xTB_ is significantly lower. Further, note that although Cu^2+^ is a d^9^ system with a comparatively simple electronic structure, isolating Cu^2+^ MOFs by the oxidation state (computed using oxiMACHINE^88^) shows that GFN1-xTB underestimates their band gaps similar to the Cr/Mn/Fe/Co/Ni subset (MAE 0.63 eV). Despite the limitations, this methodology can provide a computationally efficient framework for large-scale screening of electronic properties of MOFs, as is explored in the following section.

### Δ-Learning High-Fidelity Band Gaps

The generated GFN1-xTB data were then used to train ML models that predict high-fidelity band gaps. ST-135 (computed with matminer^72^), APRDF, and RAC features (obtained using mofdscribe^73^) were used to encode the structures. 4714 MOFs were featurizable using RACs; thus, this set of MOFs was selected for ML training using an XGBoost model, as described earlier. The train, validation, and test sets were constructed in the ratio 8:1:1 using random splits, and mean absolute error (MAE) and coefficient of determination (*R* ^2^) computed on the test set were used as the performance measure. To obtain the optimal number of features to be used, different values of the number of top features were chosen, and models were trained based on each. The results are shown in Figure S1 for GFN1-xTB, PBE, and HSE06 band gap values as the target property. The MAE vs number of top feature curves plateau after approximately 400 features in each case. This was thus chosen as the optimal number of features for model training. Furthermore, 5-fold cross-validation was performed to compute statistics (with seeds 42, 78, 14, 115, and 173), and the same splits were chosen to compare various methods.

Parity plots comparing the true vs predicted values of GFN1-xTB, PBE, and HSE06 band gaps (for seed = 42) are also presented in Figure S1. The test-set MAE values for these individual models, trained directly on the target property, are 0.37, 0.38, and 0.48 eV for GFN1-xTB, PBE, and HSE06 band gaps, respectively. Note that, given a fixed dataset, descriptors, and ML model, the increasing error from GFN1-xTB to HSE06 is also consistent with the increasing complexity and computational cost of the electronic structure methods, reflecting the challenge of accurately learning high-fidelity quantum chemical properties. We also verified that model performance is robust to the relaxation approach: descriptors from xTB geometries relaxed from unrelaxed structures closely match those from PBE-relaxed geometries (cosine similarities: 0.93–1.00). XGBoost models trained on either set give similar MAEs (0.89 vs 0.88 eV; see S4), implying that applying our approach to newly synthesized MOFs where PBE geometries are unavailable is feasible.

To utilize the availability of low-fidelity data, two different approaches were employed to predict the high-fidelity band gaps: first, an XGBoost model was trained with PBE band gap values as the target property (namely, direct model). The average MAE on the test set, obtained after 5-fold cross validation, using this method was found to be 0.382 eV, along with an *R* ^2^ of 0.651. Second, a Δ-learning model was trained, i.e., a model that uses Δ as the target quantity, where Δ is the difference between the band gaps given by GFN1-xTB and DFT in this work. After training an XGBoost model, it is applied to predict the Δ values for the test set. The DFT band gaps for the test set were then reconstructed with this correction to the GFN1-xTB band gaps. For predicting PBE band gaps, this approach led to an average MAE of 0.237 eV, i.e., a 38% decrease in comparison with the direct model. This is illustrated using parity plots in Figure 4a,b. The *R* ^2^ value also improved to 0.829. Similar models were trained with HSE06 band gaps as the target property, and the same trend was observed: the average MAE using the direct model was found to be 0.486 eV (with an *R* ^2^ of 0.598) and that with the Δ-learning model was found to be 0.304 eV (with an *R* ^2^ of 0.829), a 37% decrease (illustrated in Figure 4c,d). This significant improvement obtained using Δ-learning shows how low-fidelity low-cost data can be exploited to train ML models with high accuracy for porous materials.

**Figure 4:**
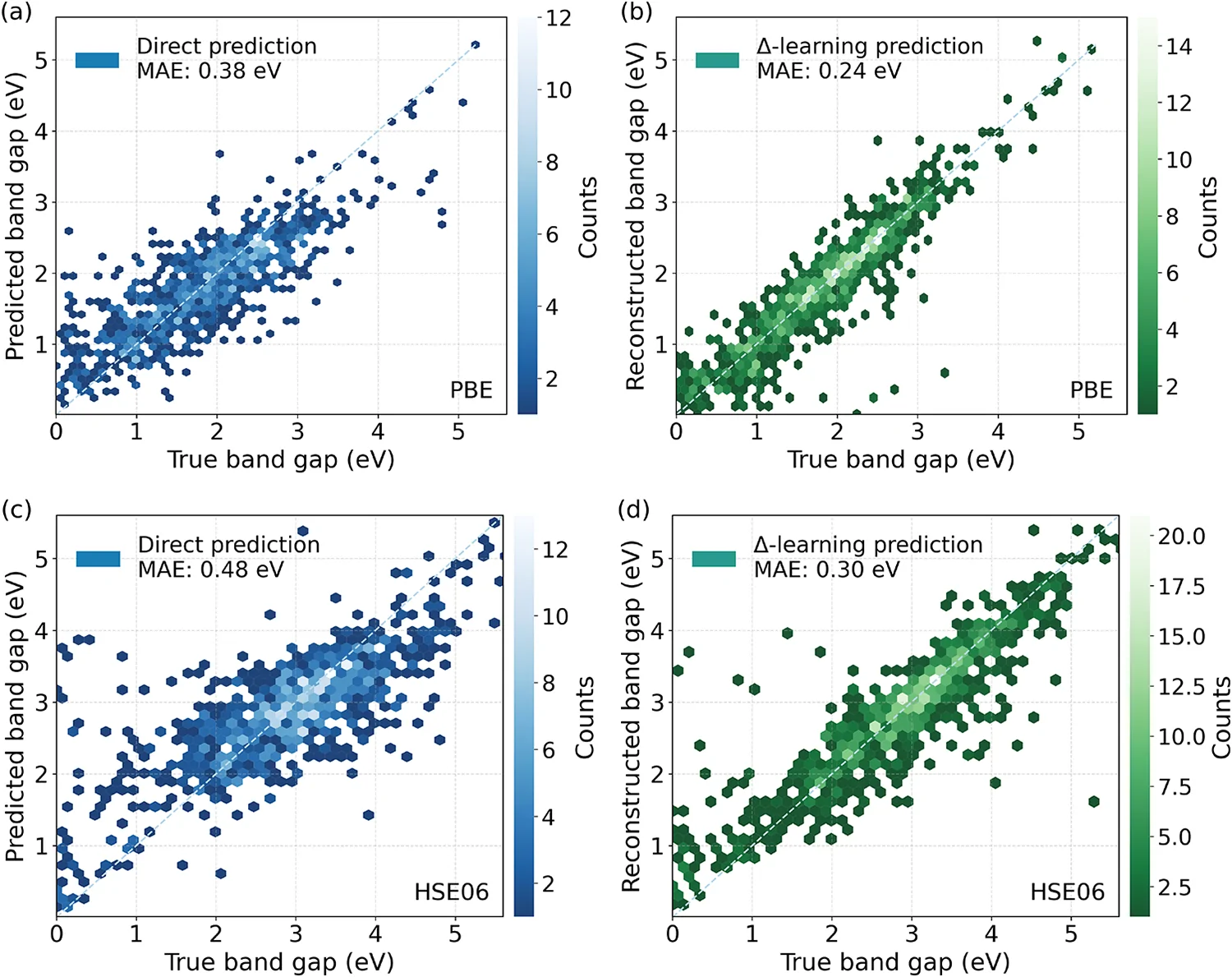
(a) Parity plot comparing true PBE band gaps of the test set with predicted band gap values obtained by training an XGBoost model using the PBE values (b) and with reconstructed band gap values obtained by training a Δ-learning XGBoost model using the GFN1-xTB and PBE band gaps. (c) Parity plot comparing true HSE06 band gaps of the test set with predicted band gap values obtained by training an XGBoost model using the HSE06 values (d) and with reconstructed band gap values obtained by training a Δ-learning XGBoost model using the GFN1-xTB and HSE06 band gaps.

It is also noteworthy that the Δ-learning model outperforms direct models despite the underestimated band gaps in the training data predicted for MOFs with transition metals. To investigate this further, we analyze the model performances specifically on the open-shell systems from the test set. This analysis revealed that the MAE obtained using the Δ-learning model (0.28 eV) is lower than that with the direct model (0.32 eV) for predicting PBE band gaps on the open-shell systems. For HSE06 band gaps, both models perform essentially the same, implying that the Δ-learning model is generalizable across various MOF types and that the underestimated band gap do not negatively impact the model performance. These results are presented in Figure S7.

We also benchmarked XGBoost against other ML methods such as Random Forest, Ridge regression, Support Vector Regression, and *K*-Nearest Neighbors, for both direct and Δ-learning prediction of PBE and HSE06 band gaps. XGBoost achieved the lowest MAE and highest *R* ^2^ overall, and the Δ-learning advantage was consistent across all model types, confirming that it is not specific to XGBoost (see S6). Finally, our models were also compared to two methods employing deep learning architectures, MOFTransformer^54^ and CGCNN,^77^ fine-tuned on our custom dataset. The results, presented in Table 1, show that the descriptor-based XGBoost model performs comparably to these methods, despite relying on engineered features rather than end-to-end learned representations, indicating that carefully designed descriptors can still capture structure–property relationships effectively. Moreover, the Δ-learning approach using xTB data significantly improves predictive accuracy and also demonstrates greater robustness across the dataset by outperforming both CGCNN and MOFTransformer. Over the 5-fold cross validation splits, the Δ-learning strategy also has low standard deviation as compared to other methods, making it consistent across various train-test splits (the individual performance metrics for each seed are provided in S7). Finally, the consistent high *R* ^2^ values, compared to the deep-learning models, show the effectiveness of this approach, reinforcing the impact of our contribution.

**Table 1:** Comparison of Different Methods for Predicting Band Gaps Using PBE and HSE06 Functionals^a^

	*E*_g,PBE_			*E*_g,HSE06_		
method	MAE (eV)	Std (eV)	*R* ^2^	MAE (eV)	Std (eV)	*R* ^2^
XGBoost^b^	0.382	**0.006**	0.651	0.486	0.022	0.598
xTB-Δ_XGBoost_ ^b^	**0.238**	0.011	**0.823**	**0.305**	**0.008**	**0.829**
MOFTransformer^54^	0.407	0.012	0.592	0.532	0.033	0.505
CGCNN^77^	0.356	0.020	0.549	0.458	0.013	0.451

a Metrics shown are mean absolute error (MAE) in eV along with its standard deviation (Std) and coefficient of determination (*R* ^2^), evaluated on the test set and averaged over five different cross-validation runs. The best performer is highlighted in bold.

b Models developed in this work.

## Discussion

In this work, a Δ-learning approach is implemented to train ML models that predict band gaps of metal–organic frameworks with high accuracy. The semiempirical GFN1-xTB method is utilized to compute electronic band gap for a dataset of ≈5000 MOFs. It is found that GFN1-xTB approximates the band gap well in comparison with DFT at the PBE level of theory (MAE = 0.37 eV). Further, the trend between band gaps obtained using GFN1-xTB and HSE06 is similar to the trend between PBE and HSE06 band gaps for MOFs. This study demonstrates that GFN1-xTB can be used to predict the band gaps of MOFs with reasonable accuracy. Note that PBE, as a GGA-level functional, is known to systematically underestimate band gaps due to residual self-interaction error. Accordingly, the PBE-GFN1-xTB comparison serves to validate that the semiempirical method captures the qualitative trends across the dataset. It is important to note that although GFN1-xTB performs well for most MOFs, it is unable to capture the electronic behavior of MOFs with transition metals accurately. This is likely due to the presence of open-shell electronic states in these materials, which are difficult to capture using semiempirical methods.

The computed GFN1-xTB band gaps are used along with reference HSE06 (and PBE) band gaps to train Δ-learning models. We have demonstrated that Δ-learning using low-cost GFN1-xTB band gaps enables accurate prediction of high-fidelity (HSE06) band gaps for MOFs. By training XGBoost models on the difference between GFN1-xTB and reference DFT values, Δ-learning not only outperforms direct prediction models, it also achieves a significantly higher accuracy than deep-learning benchmarks such as MOFTransformer and CGCNN, despite using tabular descriptors rather than graph-based or transformer architectures. Importantly, the Δ-learning correction partially recovers accuracy for the transition metal subset, suggesting that the model learns to compensate for errors introduced by the spin-restricted implementation. This work shows that taking advantage of computationally inexpensive semiempirical methods like GFN1-xTB and learning a correction to it to predict high-quality HSE06 band gaps is extremely beneficial.

The Δ-learning model developed in this work can be applied to unlabeled MOF datasets^28^ and provide significantly more accurate HSE06 band gap predictions than direct ML models trained solely on DFT data. The analyses presented in this work also suggest that in general, and especially for MOFs without transition metals, GFN1-xTB can be used to compute electronic properties of large MOF datasets, which can then be leveraged in advanced ML techniques and to assist generative AI approaches for generating novel MOFs with desired electronic properties. In this context, GFN1-xTB serves as a scalable surrogate model, enabling rapid screening and data generation that would otherwise be computationally prohibitive using DFT. The efficient use of large, inexpensive datasets for training predictive models is useful in low-data regimes. Finally, for MOFs without strongly correlated metals, GFN1-xTB provides a reliable baseline, enabling scalable screening and potential integration with generative design workflows. Such integration can accelerate inverse design by combining fast electronic property estimation with generative models, supporting the discovery of candidate MOFs with targeted electronic properties. Future work could also extend this framework to spin-unrestricted semiempirical methods, which may further improve accuracy for open-shell transition metal MOFs and broaden the applicability of our approach.

## Supplementary Material



## Data Availability

The custom Python code developed to automate the xTB calculations for MOFs is publicly available on GitHub: github.com/AshnaJose/MOF-xTB and on Zenodo at 10.5281/zenodo.21339238. The repository also includes the model training and evaluation code, the computed xTB band gaps for the 4941-MOF dataset, and the descriptors generated for this dataset to train the XGBoost models in this work.

## Notes

Feature selection results and model training details; error estimation for GFN1-xTB computed band gaps; ablation studies on relaxation strategies including chemically diverse subset selection and xTB optimization starting from experimental CIFs; descriptor similarity analysis between relaxation schemes; additional ML benchmarking; and detailed performance metrics (PDF)
