# Supplementary material for: GFN1-xTB-Assisted Machine Learning for Electronic Structure Screening of Metal–Organic Frameworks
Source: J Chem Theory Comput. 2026 Aug 11;22(16):8531–40. doi: 10.1021/acs.jctc.6c00979 (PMC13523700; doi:10.1021/acs.jctc.6c00979)
Supplement: Supplementary file 1 [file ct6c00979_si_001.pdf]

Supplementary Information to "*GFN1-xTB-Assisted Machine Learning for Electronic Structure Screening of Metal-Organic Frameworks*"

Ashna Jose* and Aron Walsh*

Department of Materials, Imperial College London, London SW7 2AZ, United Kingdom

E-mail: a.jose@imperial.ac.uk; a.walsh@imperial.ac.uk

Contents

S1 Feature selection and model training **S3**

S2 Error estimation for GFN1-xTB computed band gaps **S4**

S3 Ablation studies considering relaxation strategies **S5**

S3.1 Chemically diverse subset S5

S3.2 GFN1-xTB geometry optimisation starting from experimental CIFs S6

S4 Model performance dependence on relaxation approach **S8**

S5 Δ-Learning high fidelity band gaps **S11**

S6 Additional ML benchmarking **S12**

S7 Detailed performance metrics for XGBoost, MOFTransformer and CGCNNS15

S1 Feature selection and model training

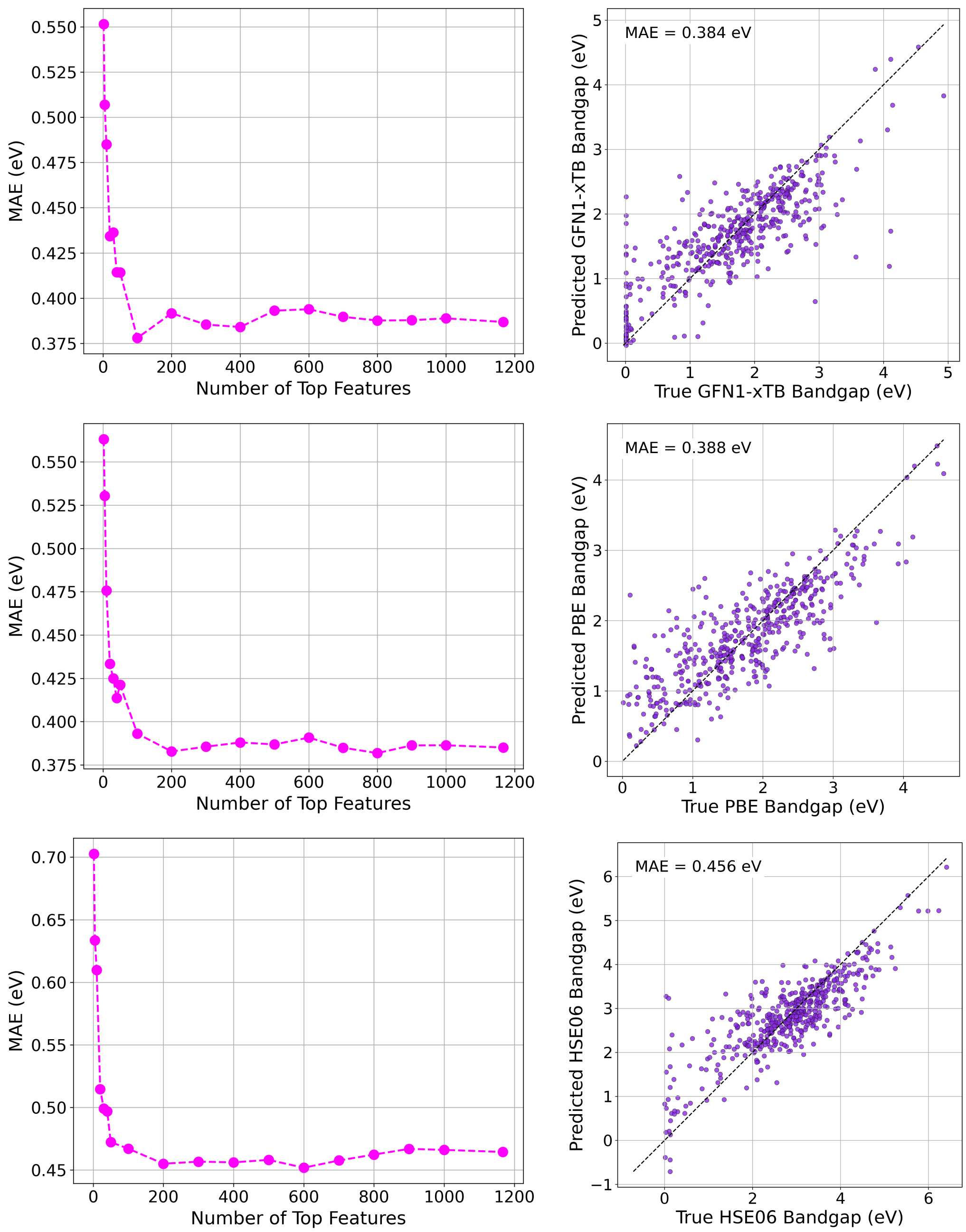

Figure S1: Left: Plots showing variation of MAE with the number of features to predict GFN1-xTB, PBE and HSE06 band gaps, respectively. Features are added in their decreasing order of importance. Right: Parity plots comparing the true GFN1-xTB, PBE and HSE06 band gaps for the test set with predicted band gaps using an XGBoost model trained with the respective band gaps as the targets (direct prediction), using the top 400 features. $y = x$ line is shown for reference. All the plots correspond to the train-test split with seed = 42.

S2 Error estimation for GFN1-xTB computed band gaps

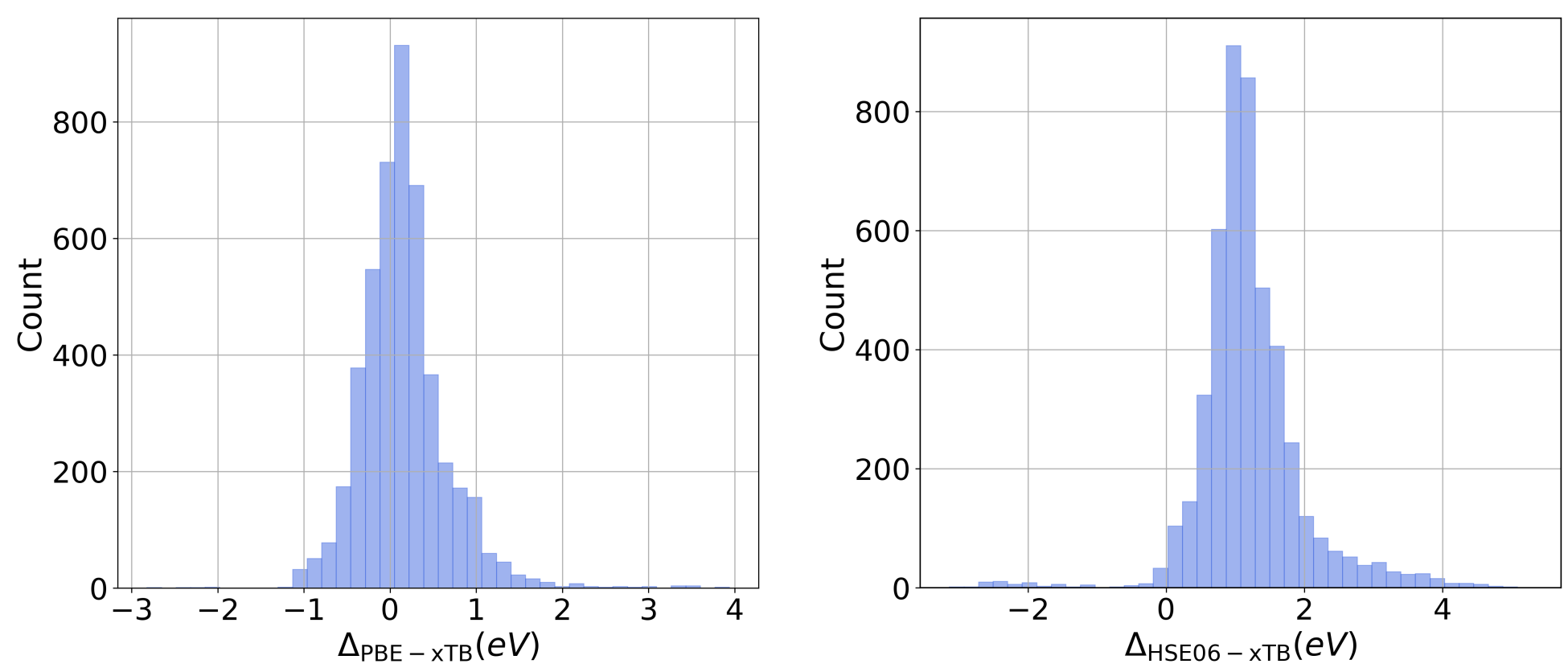

Figure S2: Histograms showing the distribution of $\Delta_{PBE-xTB}$ (left) and $\Delta_{HSE06-xTB}$ (right).

Figure S2 shows the distribution of the errors between the computed GFN1-xTB band gaps and the reference DFT band gaps (PBE and HSE06). It can be observed that GFN1-xTB approximates PBE values well, with an MAE of 0.37 eV. It underestimates HSE06 in general, with an MAE of 1.26 eV.

S3 Ablation studies considering relaxation strategies

S3.1 Chemically diverse subset

To asses if the subset selection criterion affects the variation between GFN1-xTB-relaxed vs. non-relaxed band gaps, the band gap stratification used in the main text was replaced with a chemistry-driven selection approach. We used the ST-135 stoichiometric descriptors that were computed for all structures in our dataset for this selection as these features encode element-level statistics that collectively capture the bulk chemical composition of each MOF. We then applied a farthest-point sampling (FPS) on the standardised ST-135 feature space to select a chemically diverse subset. FPS iteratively selects the structure furthest (in Euclidean distance) from all previously selected structures, ensuring that the resulting subset has maximum coverage of the chemical feature space. 500 MOFs were selected using this method, and GFN1-xTB band gaps were computed for these with relaxation (starting from PBE optimised structures, using the same settings as described in the main text). The distribution of the dominant metals in each MOF shown in Figure S3 for the QMOF-4941 dataset vs the chemically diverse-500 subset demonstrates that the subset is chemically diverse. Note that Zn, Cu, and Al are most frequent (broadly representative of the full QMOF distribution).

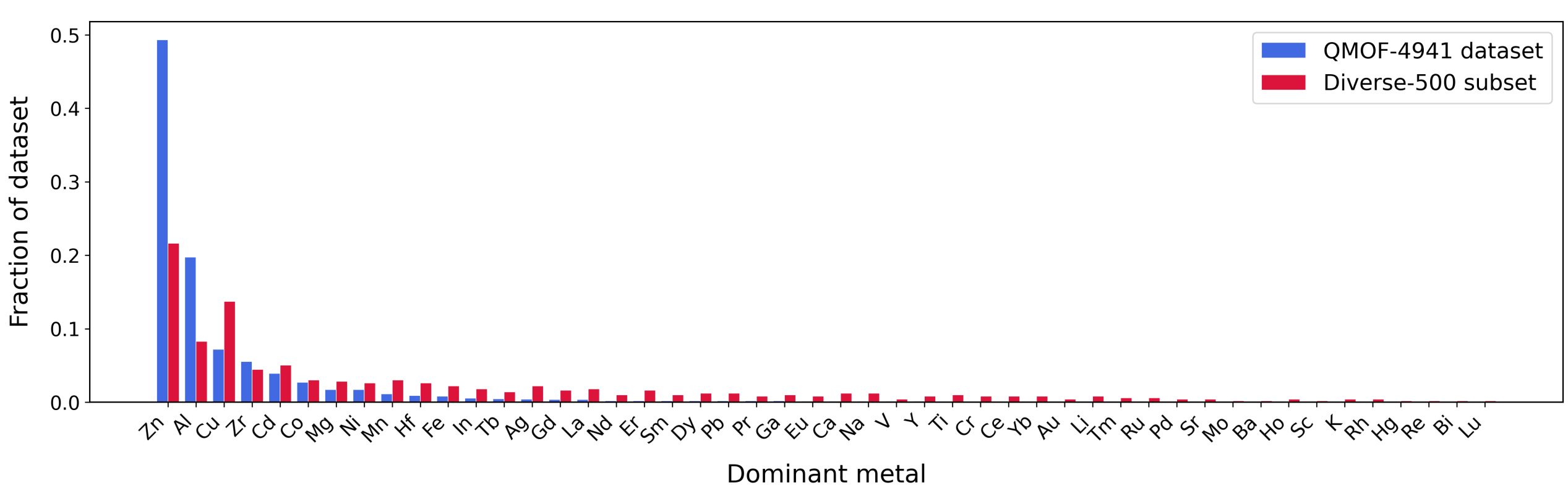

Figure S3: Metal-distributions of the QMOF-4941 dataset, compared to that of the chemically-diverse FPS-selected-500 subset.

The comparison of band gaps obtained using the pre-relaxed structures vs xTB-relaxed structures for the chemically diverse subset are shown in Figure S4. Similar to the results from the subset selected using band gap diversity, the kernel density estimate (KDE) distributions indicate that relaxation with GFN1-xTB underestimates the band gaps in some regions, while slightly overestimates in others, although the overall trend remains consistent. Thus, regardless of the choice of subset, our analysis of the band gaps obtained with and without xTB relaxation holds.

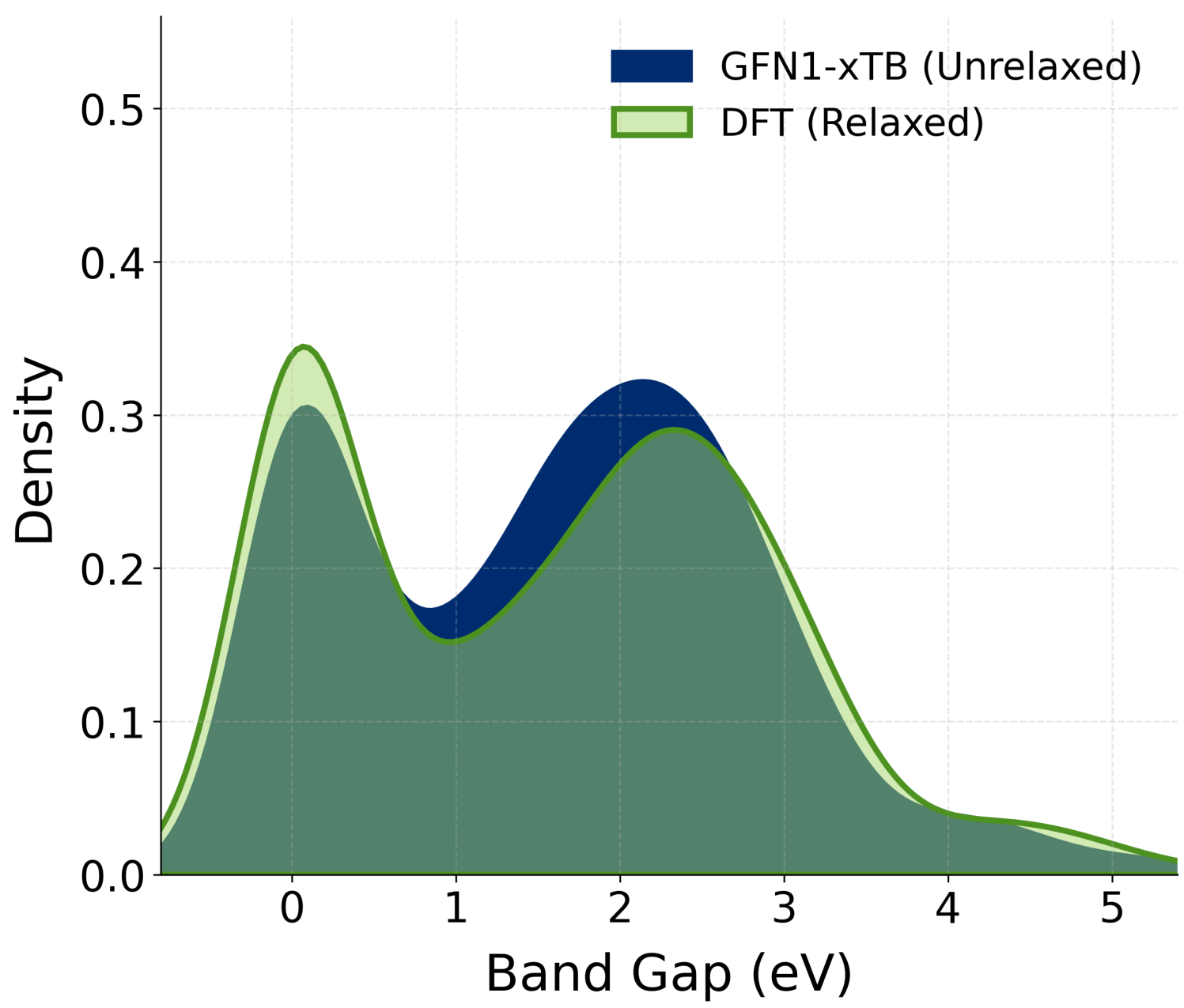

Figure S4: Band gap distributions of 500 chemically diverse MOFs obtained from unrelaxed and relaxed GFN1-xTB calculations using kernel density estimates (resulting in the tail that extends below 0 eV).

S3.2 GFN1-xTB geometry optimisation starting from experimental CIFs

Additional tests were also performed GFN1-xTB geometry optimisation on a subset of MOFs starting from the unrelaxed experimental CSD structures as provided in the CoRE MOF 2019 database.[1] These experimental CIFs represent the structures with free solvent removed but no prior DFT relaxation applied. A subset of 93 MOFs was chosen from the 500 chemically

diverse MOFs selected using FPS. This set was chosen such that it is chemically diverse, with number of atoms less than 200, to reduce computational complexity for this ablation study. Of the 93 structures, 84 converged successfully (the others did not converge within 500 geometry steps, and these structures were excluded from subsequent analysis).

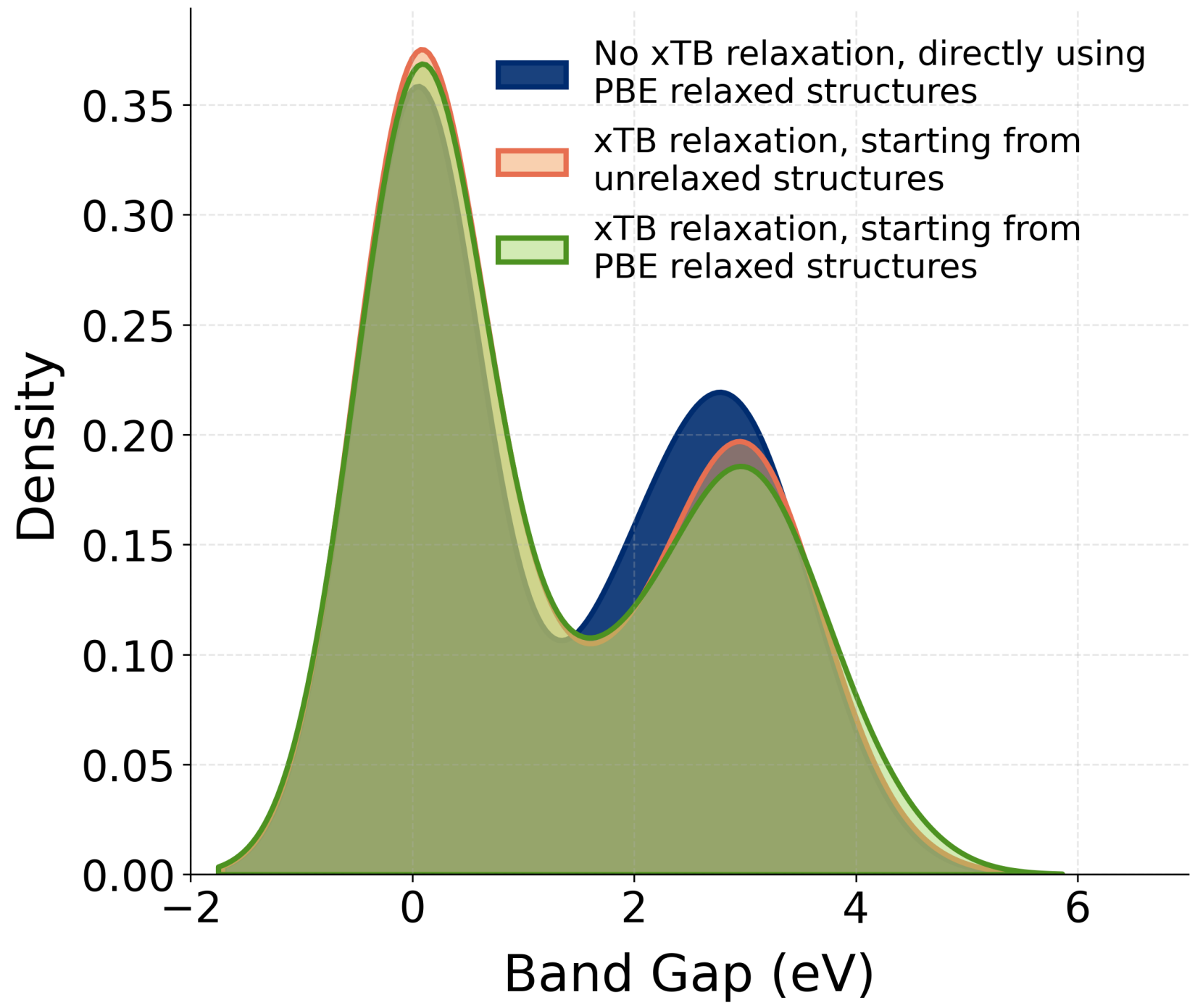

Figure S5: Band gap distributions of $\sim$ 80 chemically diverse MOFs obtained using various relaxation strategies. The distributions are represented using kernel density estimates (resulting in the tail that extends below 0 eV).

Figure S5 shows the band gap distributions of the $\sim$ 80 chemically diverse MOFs obtained using various relaxation strategies. The distribution of band gaps obtained using xTB relaxation, starting from completely unrelaxed structures matches well with the one obtained using xTB relaxation applied to PBE relaxed structures from the QMOF database. This is also confirmed from the parity plot shown in Figure S6 (b) which shows a very low MAE (0.07 eV) between the two cases. Thus, band gap values predicted from GFN1-xTB are not very sensitive to the relaxation choice, and starting from experimental unrelaxed structures, GFN1-xTB relaxes to a similar geometry that PBE does.

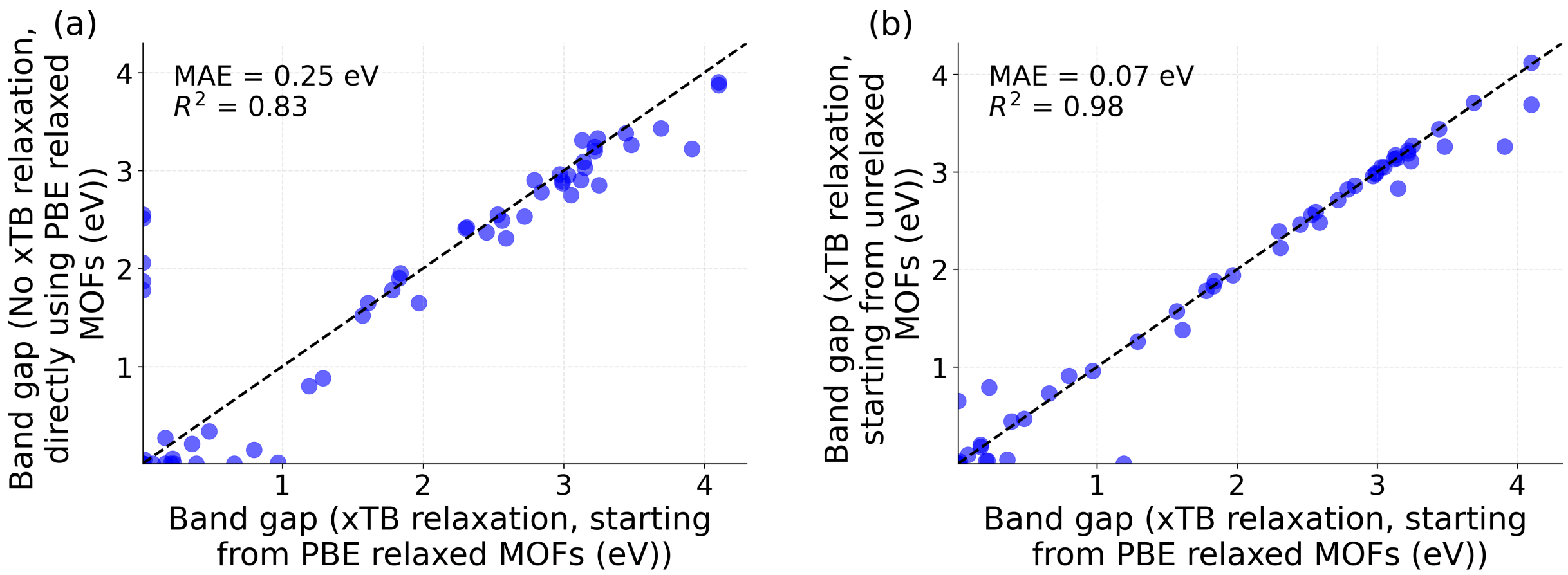

Figure S6: Parity plots comparing band gaps obtained using various relaxation strategies: (a) using PBE relaxed MOFs, with xTB relaxation vs no relaxation and (b) xTB relaxation starting from PBE relaxed vs unrelaxed MOFs.

S4 Model performance dependence on relaxation approach

To asses potential change in model performance that may be caused due to xTB vs PBE relaxations, we first performed a comparison of the three descriptors between the GFN1-xTB geometries relaxed starting from the PBE-relaxed QMOF geometries vs the unrelaxed geometries for the set of 83 MOFs discussed previously. For each pair of MOFs, we computed the descriptors used to train our models: ST-135, RACs and APRDFs for both geometries and quantified the similarity between the descriptors using the cosine similarity metric:

$$\text{cosine similarity} = \frac{\mathbf{x}_{\text{PBE}} \cdot \mathbf{x}_{\text{unrelaxed}}}{||\mathbf{x}_{\text{PBE}}|| \cdot ||\mathbf{x}_{\text{unrelaxed}}||}, \tag{1}$$

where $\mathbf{x}_{\text{PBE}}$ and $\mathbf{x}_{\text{unrelaxed}}$ are descriptor vectors for the features obtained from PBE-relaxed QMOF geometries (subsequently relaxed by xTB) and unrelaxed geometries (also subsequently relaxed by xTB) respectively. A value of 1.0 indicates identical feature vectors, while values approaching 0 indicate maximally different feature vectors. The results are summarised below:

- **ST-135 (Meredig compositional features, 135 dimensions):** As these features depend only on elemental composition and stoichiometric fractions, the cosine similarity between ST-135 vectors was 1.00 for all MOFs, confirming identical features when the chemical formula is preserved.

- **RACs (Revised Autocorrelation descriptors, 384 dimensions):** RACs encode the local environment around metal nodes and organic linkers through graph-based autocorrelation of atomic properties (electronegativity, nuclear charge, topology etc.) up to graph distances of 1-3 bonds. They can thus be sensitive to local coordination geometry and bond connectivity. The mean cosine similarity across the MOF pairs was found to be $\mathbf{0.997 \pm 0.005}$, with a minimum of 0.964. Thus, the local bonding is essentially unchanged between the two relaxation schemes, and the metal coordination environments, linker connectivity, and local chemical environments are well preserved.

- **APRDFs (Atomic-Property-Weighted Radial Distribution Functions, 648 dimensions):** APRDFs encode the distribution of interatomic distances weighted by products of atomic properties (electronegativity, atomic mass, atomic radius) at discrete distance bins from 0 to ∼20 Å. APRDFs are sensitive to the continuous distribution of all pairwise distances in the structure, making them more responsive to geometry changes. The mean cosine similarity across the MOF pairs was found to be $\mathbf{0.929 \pm 0.068}$, with a minimum of 0.644. While lower than the RAC similarity, a similarity of 0.929 indicates strong preservation of the overall features. The most changed APRDF features were those encoding atomic mass product distributions, which may be due to redistribution of interatomic distances during relaxation without fundamental changes to the structural topology.

From the above results, we can confirm that starting from unrelaxed geometries and relaxing using xTB gives descriptors similar to those obtained using PBE geometries. To analyse its effect on model performance, we trained XGBoost models on the combined ST-

135 + RAC + APRDF feature set extracted from the xTB-relaxed geometries (starting from unrelaxed structures), and compared performance against a model trained on the same features extracted from xTB-relaxed geometries (starting from the PBE optimised OMOF geometries) for this subset of MOFs. As the dataset size is very small, feature selection was performed by fitting an XGBoost model on the training set and retaining the top 50 features. Models were evaluated using 5-fold cross-validation, however, note that due to the small sample size ($\sim$ 80 MOFs), these results are interpreted as relative comparisons rather than absolute performance estimates.

The overlap between the top-50 most important features for the two cases was approximately 60–70%, indicating that similar chemical descriptors drive band gap prediction regardless of the starting geometry. The test-set MAE was 0.891 eV for the case where starting geometries were unrelaxed, while that using the PBE-relaxed geometries as the starting point, the MAE was 0.884 eV. Even though this analysis is performed on a very small dataset (resulting in a high MAE), the overall trend shows that the results are similar and largely agnostic to the relaxation scheme. This confirms that model performance does not degrade when features are extracted from xTB-optimised structures starting from experimental CIFs, supporting the practical application of our approach for newly synthesised MOFs where PBE-optimised geometries are not available.

S5 Δ-Learning high fidelity band gaps

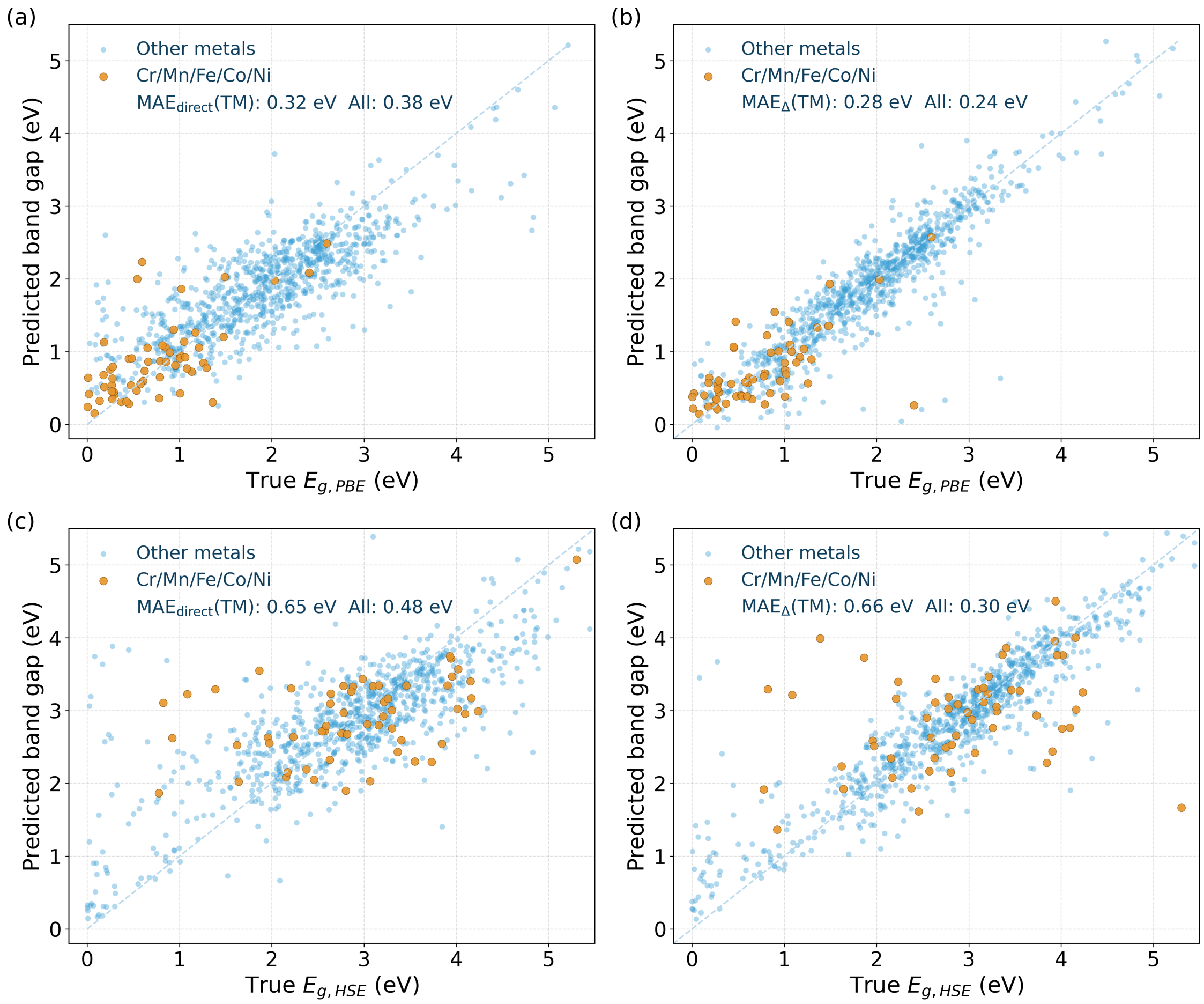

Figure S7: Parity plots comparing direct and Δ-learning predictions against true DFT band gaps for (a,b) PBE and (c,d) HSE06 levels of theory for the test set. In each panel, MOFs containing open-shell transition metals (Cr, Mn, Fe, Co, Ni) are highlighted in orange, while all other MOFs are shown in blue. Their respective MAE values are reported as well.

S6 Additional ML benchmarking

We also benchmarked our models against alternative regression algorithms: Random Forest, Ridge Regression, Support Vector Regression (SVR with RBF kernel, $C = 10$, $\epsilon = 0.1$), and K-Nearest Neighbours (KNN, $k = 5$). All models were evaluated using the same formalism as the main paper: five independent train/test splits with random seeds 42, 78, 14, 115, and 173 (80:20 split ratio), with feature selection performed independently on each seed's training set (top 400 features chosen as before), and performance averaged across seeds to report mean MAE, standard deviation, and R^2. Both direct and Δ-learning predictions were evaluated for PBE and HSE06 targets.

The results presented in Table S1 show that overall, XGBoost achieves the lowest MAE and highest R^2, for both direct and Δ-learning, for PBE and HSE06 targets, justifying its selection in our work. Further, note that the Δ-learning approach consistently outperformed direct prediction for all model types, demonstrating that the advantage of Δ-learning is not model-dependent and is not specific to XGBoost. The Tree-based ensemble methods performed comparably to each other but consistently below XGBoost, while other models showed a higher MAE. Thus, for the data regime used in this work, XGBoost models achieve the best performance. The results of this analysis (MAE and R^2) are also presented in Figure S8 (for predicting PBE band gaps) and S9 (for predicting HSE06 band gaps).

Table S1: Comparison of different methods for predicting PBE and HSE06 band gaps. Metrics shown are Mean Absolute Error (MAE) in eV along with its standard deviation (Std), and coefficient of determination (R^2), evaluated on the test set and averaged over five different cross-validation runs. Results are shown for both direct prediction and Δ-learning.

**PBE**	Direct			Δ-learning		
Method	MAE (eV)	Std (eV)	R^2	MAE (eV)	Std (eV)	R^2
XGBoost	0.382	0.006	0.651	0.238	0.011	0.823
Random Forest	0.469	0.013	0.522	0.272	0.008	0.792
Ridge	0.470	0.015	0.451	0.290	0.010	0.742
SVR	0.356	0.009	0.639	0.233	0.008	0.827
KNN	0.491	0.018	0.450	0.268	0.012	0.783
**HSE06**	Direct			Δ-learning		
XGBoost	0.486	0.022	0.598	0.305	0.008	0.829
Random Forest	0.576	0.013	0.502	0.395	0.018	0.736
Ridge	0.595	0.015	0.411	0.401	0.014	0.683
SVR	0.501	0.019	0.564	0.329	0.014	0.769
KNN	0.613	0.015	0.400	0.396	0.025	0.676

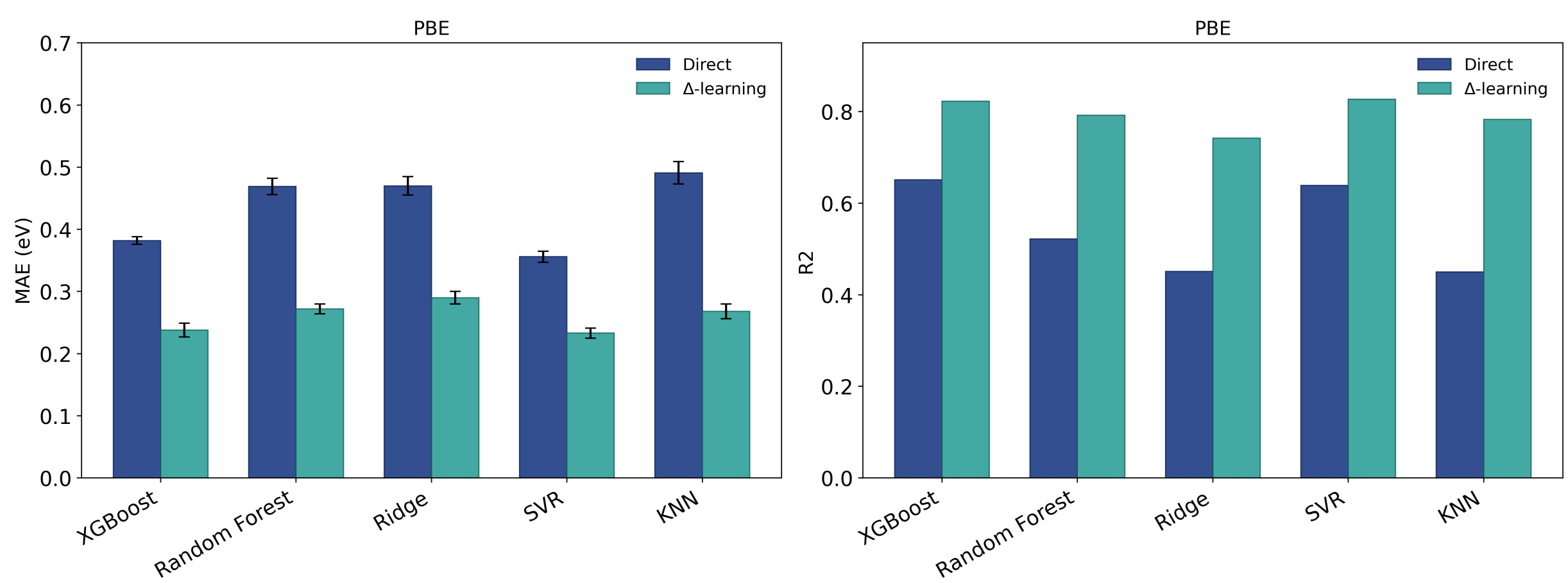

Figure S8: Benchmarking of regression models for PBE band gap prediction using direct and Δ-learning frameworks. Mean absolute error (MAE, eV) and coefficient of determination (R^2), averaged over five independent train/test splits (seeds 42, 78, 14, 115, 173) are shown. Error bars denote standard deviation across seeds. Top 400 descriptors from the combined ST-135, RAC, and APRDF feature set were used to train the models.

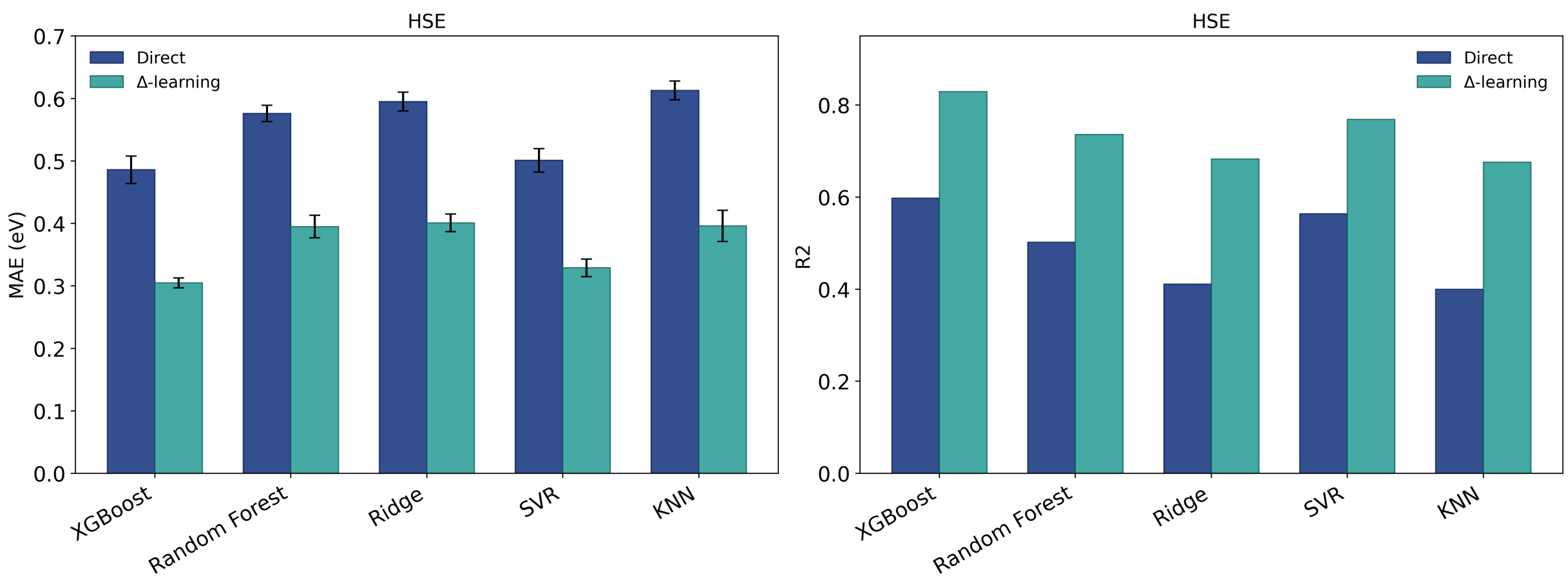

Figure S9: Benchmarking of regression models for HSE06 band gap prediction using direct and Δ-learning frameworks. Mean absolute error (MAE, eV) and coefficient of determination (R^2), averaged over five independent train/test splits (seeds 42, 78, 14, 115, 173) are shown. Error bars denote standard deviation across seeds. Top 400 descriptors from the combined ST-135, RAC, and APRDF feature set were used to train the models

S7 Detailed performance metrics for XGBoost, MOF-Transformer and CGCNN

Table S2: XGBoost performance across different random seeds. MAE values are reported in eV.

Seed	MAE^{PBE}_{dir}	$R^{2,PBE}_{dir}$	MAE^{PBE}_{Δ}	$R^{2,PBE}_{\Delta}$	MAE^{HSE}_{dir}	$R^{2,HSE}_{dir}$	MAE^{HSE}_{Δ}	$R^{2,HSE}_{\Delta}$
42	0.388	0.659	0.250	0.805	0.457	0.683	0.317	0.821
78	0.384	0.629	0.238	0.831	0.497	0.496	0.307	0.810
14	0.383	0.635	0.218	0.875	0.481	0.621	0.304	0.816
115	0.371	0.680	0.241	0.795	0.475	0.625	0.298	0.825
173	0.385	0.654	0.242	0.811	0.522	0.584	0.297	0.876
**Avg**	0.382	0.651	0.238	0.823	0.486	0.598	0.305	0.829
**Std**	0.006	0.019	0.011	0.029	0.022	0.068	0.008	0.025

Table S3: CGCNN performance across different random seeds. MAE values are reported in eV.

Seed	MAE^{PBE}	$R^{2,PBE}$	MAE^{HSE}	$R^{2,HSE}$
42	0.368	0.463	0.439	0.480
78	0.374	0.400	0.465	0.299
14	0.320	0.619	0.471	0.537
115	0.356	0.608	0.452	0.485
173	0.363	0.657	0.465	0.453
**Avg**	0.356	0.549	0.458	0.451
**Std**	0.020	0.103	0.013	0.087

Table S4: MOFTransformer performance across different random seeds. MAE values are reported in eV.

**Seed**	$\mathbf{MAE^{PBE}}$	$\mathbf{R^{2,PBE}}$	$\mathbf{MAE^{HSE}}$	$\mathbf{R^{2,HSE}}$
42	0.401	0.629	0.525	0.575
78	0.416	0.570	0.552	0.399
14	0.392	0.568	0.532	0.503
115	0.400	0.622	0.481	0.568
173	0.424	0.572	0.570	0.481
**Avg**	0.407	0.592	0.532	0.505
**Std**	0.012	0.026	0.033	0.067

References

(1) Chung, Y. G.; Haldoupis, E.; Bucior, B. J.; Haranczyk, M.; Lee, S.; Zhang, H.; Vogiatzis, K. D.; Milisavljevic, M.; Ling, S.; Camp, J. S.; Slater, B.; Siepmann, J. I.; Sholl, D. S.; Snurr, R. Q. Advances, Updates, and Analytics for the Computation-Ready, Experimental Metal–Organic Framework Database: CoRE MOF 2019. *Journal of Chemical & Engineering Data* **2019**, *64*, 5985–5998.
